# Children’s Empathy and Their Perception and Evaluation of Facial Pain Expression: An Eye Tracking Study

**DOI:** 10.3389/fpsyg.2017.02284

**Published:** 2017-12-22

**Authors:** Zhiqiang Yan, Meng Pei, Yanjie Su

**Affiliations:** Beijing Key Laboratory of Behavior and Mental Health, School of Psychological and Cognitive Sciences, Peking University, Beijing, China

**Keywords:** child, pain, empathy, facial expression, eye tracking

## Abstract

The function of empathic concern to process pain is a product of evolutionary adaptation. Focusing on 5- to 6-year old children, the current study employed eye-tracking in an odd-one-out task (searching for the emotional facial expression among neutral expressions, *N* = 47) and a pain evaluation task (evaluating the pain intensity of a facial expression, *N* = 42) to investigate the relationship between children’s empathy and their behavioral and perceptual response to facial pain expression. We found children detected painful expression faster than others (angry, sad, and happy), children high in empathy performed better on searching facial expression of pain, and gave higher evaluation of pain intensity; and rating for pain in painful expressions was best predicted by a self-reported empathy score. As for eye-tracking in pain detection, children fixated on pain more quickly, less frequently and for shorter times. Of facial clues, children fixated on eyes and mouth more quickly, more frequently and for longer times. These results implied that painful facial expression was different from others in a cognitive sense, and children’s empathy might facilitate their search and make them perceive the intensity of observed pain on the higher side.

## Introduction

A human face expressing pain may indicate the need for help or the presence of threat, to which observers should react as quickly and accurately as possible, and this highlights the importance of the capacity of “reading” pain for people. Studies have suggested important factors that would influence our reading of, as well as reactions to pain, among which an essential one is empathy (Goubert et al., 2005). It refers to the process to perceive and be responsive to others’ emotions, which enables us to react appropriately (Decety et al., 2016). Studies have found that higher empathy was associated with better performance and deeper involvement in a task of facial expression recognition (Choi and Watanuki, 2014). Despite the findings on the relationship between empathy and reading of pain in adults, little attention has been given to children. Empathy can be divided into a cognitive and an affective facet, and children’s empathy mostly takes the form of affective empathy, such as emotion contagion and emotion sharing (de Waal, 2008; Decety, 2010; Huang and Su, 2012). Here, we used eye-tracking in combination with computerized odd-one-out task and pain evaluation task to investigate the relationship between children’s empathy and their perception and evaluation of facial pain expression.

Indeed, facial expressions are one of the most important visual signals of pain (Kunz et al., 2012; Schott, 2015). Previous research has suggested that pain is a highly affective state that is accompanied by typical facial expressions (Williams, 2002; Goubert et al., 2005). As evolutionary psychologists suggest, pain may involve two functions. Pain attracts the observer’s attention and evokes approaching or avoiding behaviors in him or her (Williams, 2002); for the signal sender, pain signals threat and alerts the observer, and may evoke empathic and pro-social behaviors in the observer (Decety, 2010; Prkachin, 2011). The current research was intended to provide insight as to how children decoded visually expressed pain as a distinct emotion.

First, the facial expression of pain is characterized by some facial clues that could be detected visually and contribute to the evaluation of pain (Deyo et al., 2004; Prkachin, 2011). Deyo et al. (2004) found that 5- to 6-year old children could discriminate facial expressions of pain from neutral ones, and children were able to make better use of facial clues for this discrimination as they grew up. Other researchers also found that 3- and 12-year-old children could detect pain in others and assess pain intensity indexed by facial expression (Grégoire et al., 2016). Roy et al. (2015) employed the bubbles method and found that the facial clues of frown lines and the mouth carry the most reliable information for adults to decide whether a facial expression shows pain or not, but researchers are still far from answering the same question in children (Craig et al., 2010; Prkachin, 2011). For vulnerable children to survive, facial expressions convey some of the most salient clues that children can display to attract caregivers’ attention (Cole and Moore, 2014; Finlay and Syal, 2014). Besides, relying more on bottom-up processing, children are better subjects than adults for investigating which perceptual features of a facial expression contribute to the search and evaluation of the expression (LoBue and DeLoache, 2010; LoBue et al., 2010).

Second, some individual factors would influence how efficiently and accurately observers decode pain, such as empathy (Goubert et al., 2005; Jackson et al., 2005). Specifically, decoding capability may be related to their abilities of affective sharing and cognitive regulation, which are core components of empathy (Decety and Svetlova, 2012). As we know, perception and evaluation of pain is closely linked with empathy in adults (Grynberg and Maurage, 2014; Schott, 2015). The hypothetic theory of empathy-altruism indicates that empathy may induce altruistic motivation (Batson and Shaw, 1991; Decety et al., 2016), which in turn may be closely related to our reaction to the signals of pain. However, few studies have focused on children as to how empathy influences their recognition of pain. It is widely believed that cognitive empathy and affective empathy do not develop in parallel (Decety, 2010; Huang and Su, 2012), with affective empathy developing first or we are born with a certain degree of affective empathy (Preston and de Waal, 2002; Decety, 2010). Present researches suggest the cognitive component may be dominant in adults’ empathy (Chen et al., 2014; Cheng et al., 2014). Grégoire et al. (2016) have investigated children’s empathy for pain, but failed to find any relationship, presumably because they adapted the self-report Interpersonal Reactivity Index (Davis, 1983) to a teacher-reported format Therefore, the current study had revised the measurement of empathy with a questionnaire used widely for children, and adapted the experimental tasks for study on children.

To sum up, we designed two experiments to assess the influence of children’s empathy upon their behavioral patterns in perception and evaluation of pain shown by facial expression. In the first experiment, we planned to test how pain facial expression was perceived differently from others and what the relationship was between children’s empathy and their perception performance. A 2 (Empathy: high, low) × 4 (Facial expression type: painful, happy, sad, angry) two-factor mixed experiment was used. In the second, we planned to test what facial clues were essential for children’s evaluation of others’ pain and what the relationship was between children’s empathy and their evaluated level of pain. A 2 (Empathy: high, low) × 3 (Facial expression type: painful, sad, angry) two-factor mixed experiment was employed. We used eye-tracking devices as in previous research (Vervoort et al., 2013; LoBue et al., 2014) in order to provide data with high temporal resolution to investigate the dynamics of the attentional processing stage. We predicted that painful faces would be searched for more quickly than others and evaluated as more painful than others, and children’s empathy would be positively associated with their performance in searching, and with their perceived intensity of pain. As for attentional processing, children’s empathy would influence the later stage of attention (attentional maintenance), and facial clues of mouth and eyes would be more helpful for them to view the faces.

## Experiment 1

### Participants

Sixty-two 5- to 6-year-old children were recruited from a local kindergarten. According to the kindergarten’s official records, these children were normally developing and showed no signs of mental disease. This experiment was approved by the Ethics Committee of the School of Psychological and Cognitive Sciences at Peking University, we obtained informed consent from their guardians. In accordance with the Declaration of Helsinki, we provided parents of each participant with a written description of the experiment before it began. All parents stated in written informed consent that they allowed their child to participate. Fifteen participants did not meet the data quality criteria and were excluded (e.g., eye movements tracked < 75% of total viewing time in task) (Vervoort et al., 2013), but this exclusion did not affect the nature of the results in terms of significance and directionality. Finally, 47 children (*M*_age_ = 71.21 months, *SD*_age_ = 5.72; 21 males) were analyzed. Participants scoring on a self-reported questionnaire (see below) lower and higher than the median score formed the low and the high empathy groups, respectively. There was no difference in gender composition between the two groups [χ^2^(1, *N* = 47) = 1.53, *p* > 0.05]. According to previous research (Zaitchik et al., 2014; Freier et al., 2015; Grégoire et al., 2016), preliminary analysis revealed no significant effects of gender on either task, thus gender was not further analyzed.

### Design and Material

Differences between facial expression of pain and other emotions would contribute to the recognition of pain; therefore, we intended to address the specificity of pain by including four emotional face types. A 2 (Empathy: high, low) × 4 (Facial expression type: painful, happy, sad, angry) two-factor mixed experiment was planned.

**FIGURE 1:**
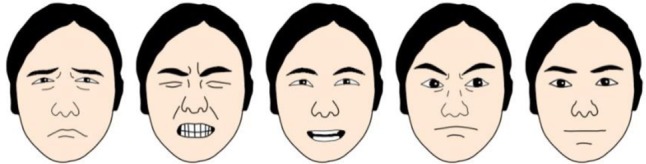
A sample set of five faces used in Experiment 1. From left to right: sad, painful, happy, angry, and neutral.

In the current study with young children, cartoon faces were preferred to real ones as the emotional information in the former was more easily accessed (Kendall et al., 2015). Thus, we collected thirteen neutral real faces as raw material from the gallery of Simon et al. (2008) and invited professional artists to convert them into cartoons with PaintTool SAI, removing any clue for gender. Then, according to results from Williams (2002) on facial action units (FAUs), we designed emotional faces that were morphed from each neutral one into the four emotional expressions, making a total of thirteen sets in five expressions (for a sample set, see **Figure 1**). Each was 200 pixels × 250 pixels, or 7.1 cm × 8.8 cm in size, and luminance was controlled. In order to evaluate the FAUs, two graduates in psychology coded them by the facial action coding system (Ekman et al., 2002, Chapter 12, p. 174, Table 1; Williams, 2002), with Cronbach’s α equal to 0.90. Next, we invited eighteen undergraduates majoring in psychology to evaluate these pictures on five dimensions with Likert scales. They were instructed to “rate how the person in the picture might feel, with respect to valence: 1 = clearly unpleasant to 9 = clearly pleasant; and arousal: 1 = highly relaxed to 9 = high level of arousal; and to “judge the type of facial expression (happy, neutral, sad, angry, and painful)” and “indicate how confident you are about your judgment on expression type: 1 = not sure to 9 = totally sure”; and lastly to “rate the intensity of each emotion in the picture (such as intensity of happiness in a happy face) from 1 = not at all to 6 = the most intense possible.” Statistics on all 65 pictures are shown in **Table 1**. Just as in previous research (Kappesser and Williams, 2002; Simon et al., 2008), significant variations were found on all dimensions. Pairwise analysis showed that painful (*M* = 4.53, *SE* = 0.19, *p*s < 0.01) and happy (*M* = 4.42, *SE* = 0.14, *p*s < 0.01) faces were greater in the intensity of emotion than angry (*M* = 3.45, *SE* = 0.21), sad (*M* = 3.67, *SE* = 0.22) and neutral (*M* = 1.71, *SE* = 0.16) ones. Happy faces (*M* = 6.88, *SE* = 0.26, *p*s < 0.001) were greater in the valence of emotion than angry (*M* = 3.54, *SE* = 0.21), sad (*M* = 3.02, *SE* = 0.22), painful (*M* = 2.87, *SE* = 0.28) and neutral (*M* = 4.93, *SE* = 0.09) ones. Happy faces (*M* = 6.48, *SE* = 0.28, *p*s < 0.01) were greater in the arousal of emotion than angry (*M* = 4.95, *SE* = 0.34), sad (*M* = 4.93, *SE* = 0.39), and neutral (*M* = 3.47, *SE* = 0.35) ones, and painful faces (*M* = 5.49, *SE* = 0.44, *p* < 0.05) greater than neutral ones (*M* = 3.47, *SE* = 0.35). Happy (*M* = 7.27, *SE* = 0.26, *p*s < 0.01) faces were associated with greater confidence in emotion judgment than angry (*M* = 5.50, *SE* = 0.43), sad (*M* = 5.25, *SE* = 0.53), painful (*M* = 4.76, *SE* = 0.46) and neutral (*M* = 5.77, *SE* = 0.36) ones. Happy (*M* = 0.99, *SE* = 0.004, *p*s < 0.01) faces had a greater hit rate than angry (*M* = 0.65, *SE* = 0.06), painful (*M* = 0.61, *SE* = 0.06) and neutral (*M* = 0.83, *SE* = 0.04) ones; sad faces (*M* = 0.92, *SE* = 0.04) had a greater hit rate than angry and painful ones. For a task taking a reasonable amount of time, we actually selected four sets of faces as task stimuli.

**Table 1:** Experimental material rating results.

	Facial expression type *M(SD)*						
	Neutral	Happy	Angry	Painful	Sad	*F*	$\eta_{p}^{2}$
Valence	4.93 (0.39)	6.88 (1.08)	3.54 (0.87)	2.87 (1.20)	3.02 (0.93)	62.93^∗∗^	0.79
Arousal	3.47 (1.50)	6.48 (1.19)	4.95 (1.45)	5.49 (1.88)	4.93 (1.65)	14.28^∗∗^	0.46
Confidence	5.77 (1.51)	7.27 (1.12)	5.50 (1.82)	4.76 (1.96)	5.25 (2.24)	18.04^∗∗^	0.52
Emotion intensity	1.71 (0.68)	4.42 (0.61)	3.45 (0.90)	4.53 (0.82)	3.67 (0.92)	51.59^∗∗^	0.75
Hit rate (%)	82.91 (16.17)	99.57 (1.81)	65.38 (27.07)	60.68 (23.58)	91.45 (16.45)	15.59^∗∗^	0.48

^∗∗^*p < 0.01. Hit rate refers to percentage of correct judgment of a given face type*.

### Apparatus and Measures

A Tobii X120 Eye-tracker (Tobii Technology AB, Sweden) was used.

The stimuli were shown on a ThundeRobot T150 computer (screen size = 15.6′′, resolution = 1600 × 900). The experiment was programmed with Matlab and Tobii SDK, and was analyzed with EveMMV toolbox (Krassanakis et al., 2014).

To measure children’s empathy, we translated the 6-item self-reported questionnaire from Zhou et al. (2003), which has been validated against various external criteria of social behavior in developmental studies (e.g., Catherine and Schonert-Reichl, 2011), and adapted the vignette empathy story task from Strayer (1993). The Empathy Continuum Scoring System developed by the latter study integrated the degree of affective sharing experienced (i.e., degree of match between one’s own emotion and that of the stimulus person) with the child’s cognitive attribution for his or her own emotions.

To develop a localized version of the self-reported empathy questionnaire, we employed two undergraduates majoring in English to translate all items into native language and another two to translate them back to English. Then two Ph.Ds. in psychology rated the similarity between the original and the reverse-translated versions, which was high at 0.88. The translated version was effectively equivalent in meaning to the original one. Participants rated how well the statements described them, such as “I feel sorry for other kids who don’t have toys and clothes,” from 1 (not like him/her), 2 (sort of like him/her), or 3 (like him/her). In vignette empathy story task, we used videotaped version of vignette stories (Wu, 2013, The Association between Oxytocin Receptor Gene and Prosocial Behavior: Role of Person and Situation Factors. Unpublished doctoral dissertation, Peking University). Participants were invited to watch four video clips. Each video clip was 1 to 3 min long, in which a kid performed some action such as singing when playing with one parent in a daily, real-life scene, and showed one type of emotion, such as happiness. Then, participants would answer four orally asked questions: (1) “How are you feeling now?”; (2) “Why do you have this feel?”; (3) “How do you think about the kid’s feel in the story?”; and (4) “Why do you think the kid would feel like that?” The answers were coded by two graduate students majoring in psychology with the empathy continuum scoring system (Strayer, 1993).

**FIGURE 2:**
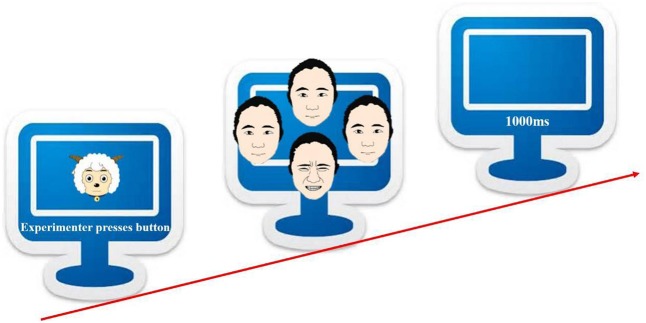
Odd-one-out schematic diagram.

The experimental task followed the odd-one-out visual search paradigm (Krysko and Rutherford, 2009). Children were seated about 60 cm from the computer screen and a chin rest was used to restrict head movement. In each trial, experiment stimuli were composed of three copies of a neutral face and one emotional facial expression (angry, happy, painful, or sad) morphed from it (see **Figure 2**). Participants should find the different one (target) by pushing the stick to the corresponding direction (forward for a target at the top, backward at the bottom, plus left and right). Eye movement pattern was collected when the participant was viewing the target picture. A gaze that remained stable within a 35-pixel radius and lasted at least 100 ms on a defined area of interest (AOI) would count as fixation to that AOI (Yang et al., 2012; Vervoort et al., 2013). Each type of emotional expression was presented in four trials, resulting in a session of sixteen trials in a random order. That meant the sequence would be re-randomized in regards to target position for each participant yet each type-position combination (e.g., painful target on the left) would appear once and only once. Reaction time (from stimulus onset until the joystick was displaced from the origin) and accuracy were recorded as dependent variables, and data above or below three SDs would be replaced by means.

As in previous research (Yang et al., 2012; Vervoort et al., 2013), three eye tracking indices were used. The first one, time to first fixation, was defined as the time it took (in ms) following the onset of a picture set to first fixation on a specific AOI. The second, fixation count, was defined as the total fixation counts that a participant made within the rectangular picture containing a particular facial expression as a stimulus. The third, total fixation time, was defined as the total duration of time in which a participant’s gaze remained fixated within the boundaries of a particular facial expression category. All three indices applied to the target face only.

### Procedure

**FIGURE 3:**
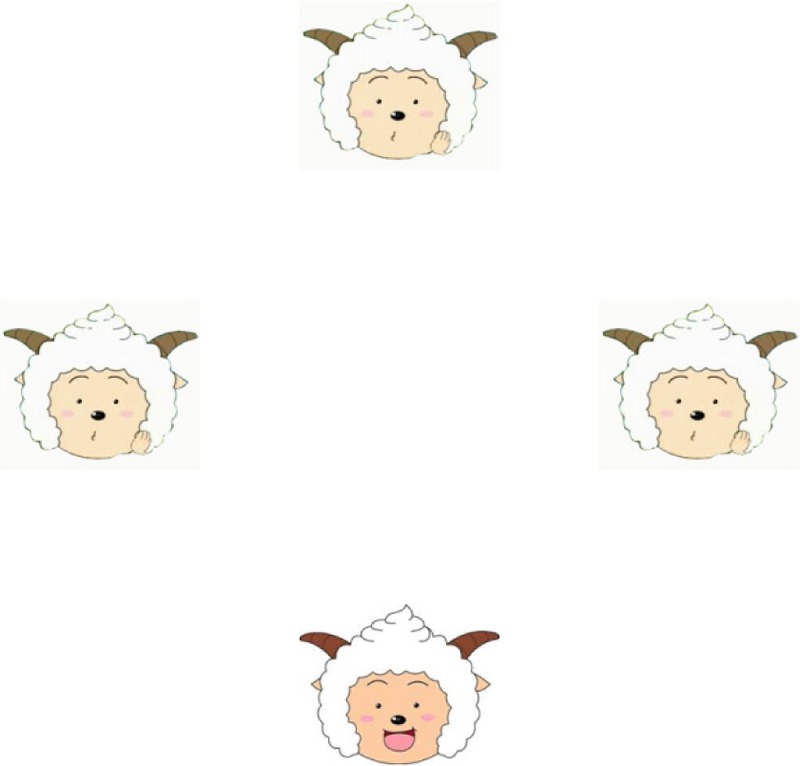
Stimuli in a sample training trial in Experiment 1, with the happy target at the bottom.

Protocol for the current study was in accordance with the ethical standards of the institutional and national research committee. Upon arrival, children were invited to complete the two measurements of empathy. Then, they would begin the odd-one-out task. They were told that the screen would present a fixation picture first, and then it would present four facial expression pictures, among which they needed to find the different one. No time limit was specified, and the stimuli would not disappear until a response was made with the joystick (see above), and the next trial would begin after an interval of 1s. Before they began the experiment, they needed to pass a calibration procedure and do eight practice trials to make sure they understood the instruction. Besides the task stimuli, another 16 internet-sourced cartoons were used as training materials (see **Figure 3**). Upon completion, they would get a toy for participation.

### Results

The measures showed good reliability, with a Cronbach’s α of 0.80 for the questionnaire (Q), and inter-rater agreement of 0.89 for the empathy story (ES) task, and the two measures were positively correlated (*r* = 0.40, *p* < 0.01). We analyzed group difference to ensure the grouping was meaningful, and there were significant differences between high empathy group (ES: *M* = 23.22, *SD* = 10.51; Q: *M* = 14.96, *SD* = 1.80) and low empathy group (ES: *M* = 15.04, *SD* = 7.50; Q: *M* = 9.25, *SD* = 1.80), with high empathy group scoring higher on both empathy story task [*t*(45) = 3.08, *p* < 0.01, Cohen’s *d* = 0.90] and self-reported empathy questionnaire [*t*(45) = 10.88, *p* < 0.001, Cohen’s *d* = 3.17] as expected.

**FIGURE 4:**
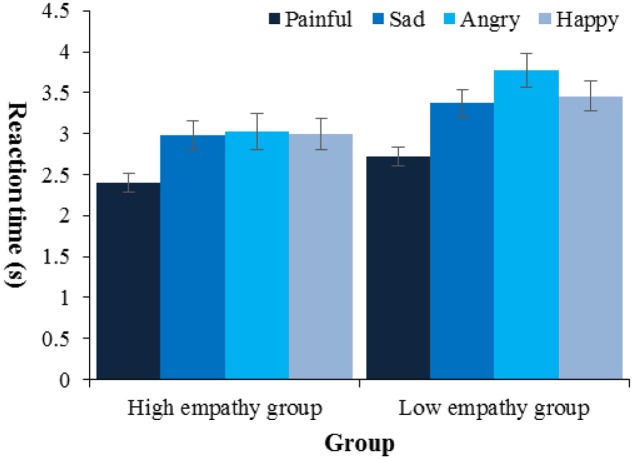
Reaction time by type of target and empathy. Error bars show standard errors in this and the following figures.

We first would like to ensure that participants did not sacrifice speed for accuracy, and a by-subject correlation analysis between reaction time and accuracy across all trials showed no significant correlation (*p* > 0.05). Then, we tested the relationship between empathy and performance in odd-one-out task. First, the difference in accuracy between high empathy (*M* = 0.93, *SD* = 0.07) and low empathy group (*M* = 0.93, *SD* = 0.06) was non-significant [*t*(45) = 0.45, *p* > 0.05, Cohen’s *d* = 0.13]. Second, a 2 (Empathy: high, low) × 4 (Facial expression type: painful, happy, sad, angry) mixed-design ANOVA was employed on reaction time (**Figure 4**). There were two main effects: facial expression type [*F*(3,135) = 16.25, *p* < 0.001, $\eta_{p}^{2}$ = 0.27] and empathy [*F*(1,45) = 6.24, *p* < 0.05, $\eta_{p}^{2}$ = 0.12]. Follow-up pairwise comparisons (All pairwise comparisons in the current article were done with Bonferroni correction) showed that children were faster when they searched for painful facial expressions (*M* = 2.56 s, *SE* = 0.08) than any other type (*p*s < 0.001), and high empathy group (*M* = 2.85 s, *SE* = 0.14) was faster than low empathy group when they searched for the odd one in a crowd of faces (*M* = 3.33 s, *SE* = 0.14, *p* < 0.05). No other effects were found. As a number of four trials for each face type were relatively few and the degree to which children’s performance stabilized within these few trials might vary with face type or empathy, we did a 2 (Empathy: high, low) × 4 (Facial expression type: painful, happy, sad, angry) mixed-design ANOVA on standard deviations of reaction time. There were two main effects: facial expression type [*F*(3,135) = 4.46, *p* < 0.01, $\eta_{p}^{2}$ = 0.09] and empathy [*F*(1,45) = 6.41, *p* < 0.05, $\eta_{p}^{2}$ = 0.13]. Follow-up pairwise comparisons showed that children performed more stably on pain (*M*_SD_ = 0.77, *SE* = 0.08) than anger (*M*_SD_ = 1.39, *SE* = 0.18, *p* < 0.05), and high empathy group (*M*_SD_ = 0.88, *SE* = 0.11) was more stable than low empathy group (*M*_SD_ = 1.26, *SE* = 0.11, *p* < 0.05).

**FIGURE 5:**
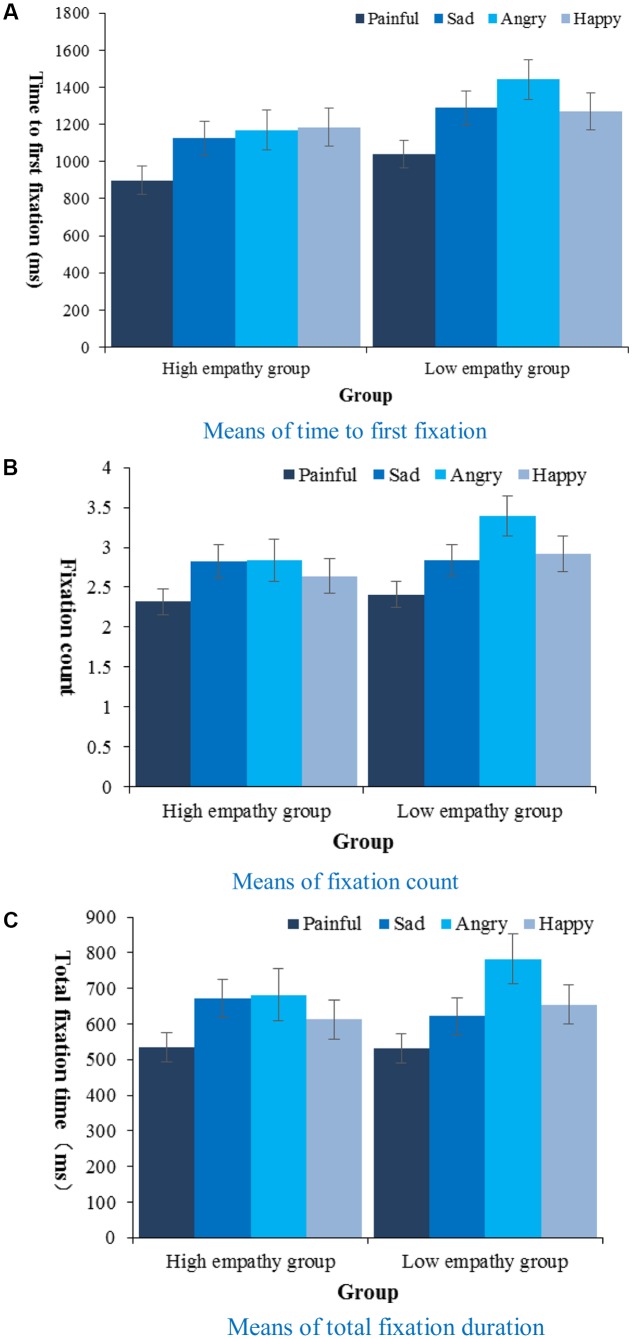
Means of time to first fixation **(A)**, fixation count **(B)**, and total fixation duration **(C)** during face searching task by type of target and empathy.

The AOI for analysis of the eye-tracking data was each picture containing the target face – all eye fixations within the frame would count. We employed 2 (Empathy: high, low) × 4 (Facial expression type: painful, happy, sad, angry) mixed-design ANOVAs on three eye-tracking indices (**Figure 5**). On the time to first fixation, there was a main effect of facial expression type [*F*(3,135) = 6.18, *p* < 0.01, $\eta_{p}^{2}$ = 0.12] and a marginal main effect of empathy [*F*(1,45) = 3.46, *p* = 0.069, $\eta_{p}^{2}$ = 0.07]. Follow-up pairwise comparisons showed that pain (*M* = 969.70 ms, *SE* = 54.27) drew attention faster than other emotions (*p*s < 0.01), and high empathy group (*M* = 1094.44 ms, *SE* = 63.47) focused marginally faster than low empathy group (*M* = 1259.72 ms, *SE* = 62.13, *p* = 0.069). On fixation count and total fixation time each, there was only a main effect of facial expression type [*F*(3,135) = 6.59, *p* < 0.001, $\eta_{p}^{2}$ = 0.13; *F*(3,135) = 7.24,*p* < 0.001, $\eta_{p}^{2}$ = 0.14, respectively]. Follow-up pairwise comparisons showed that there was a smaller fixation count on pain (*M* = 2.36, *SE* = 0.11) than on other emotions (*p*s < 0.05), and a shorter total fixation duration on pain (*M* = 532.66 ms, *SE* = 28.98) than on other emotions (*p*s < 0.05). As with reaction time, we also did 2 (Empathy: high, low) × 4 (Facial expression type: painful, happy, sad, angry) mixed-design ANOVAs on standard deviations of the three eye-tracking indices to test their stability, and found main effects of facial expression on all [*F*(3,135) = 2.71, *p* < 0.05, $\eta_{p}^{2}$ = 0.06; *F*(3,135) = 6.20, *p* < 0.01, $\eta_{p}^{2}$ = 0.12; *F*(3,135) = 3.13, *p* < 0.05, $\eta_{p}^{2}$ = 0.07, respectively]. Follow-up analysis showed that fixation count on pain (*M*_SD_ = 0.97, *SE* = 0.07, *p*s < 0.05) was associated with a smaller standard deviation than those on angry (*M*_SD_ = 1.60, *SE* = 0.15), happy (*M*_SD_ = 1.30, *SE* = 0.09) and sad (*M*_SD_ = 1.32, *SE* = 0.09) faces, and total fixation duration on pain (*M*_SD_ = 243.89 ms, *SE* = 20.84) was associated with a smaller standard deviation than that on angry (*M*_SD_ = 362.50 ms, *SE* = 38.00, *p* < 0.05).

### Discussion

Results from Experiment 1 indicated that children aged 5 and 6 could search for painful facial expression faster than for angry, happy and sad expressions. These results also agree with those found in previous research (Priebe et al., 2015). Given pain is a signal of threat and warning, participants need to notice it quickly, because doing so is essential to our health and safety (Williams, 2002; Williams and Craig, 2006). Smaller fixation count and shorter fixation duration may reflect threat aversion (LoBue, 2013; Thrasher and LoBue, 2016), for which neuroscience provided a range of evidence (Benuzzi et al., 2009; Hayes and Northoff, 2012; Kobayashi, 2012). Still, the results could be at least partly attributed to the perceptual distinctiveness of pain (Williams, 2002), and one should be cautious when interpreting them as showing differences across emotions.

Meanwhile, we found that high empathy group fixated faster than low empathy group did. This can be explained by Decety and Svetlova’s (2012) theory, which states that empathy may contribute to affective sharing and emotion recognition, or in other words, empathy would help improve our performance in emotion recognition. For example, Balconi and Canavesio (2016) have showed that empathy could affect adults’ face detection performance and attentional process. Some researchers also have found that empathy is positively related to performance in emotion recognition, an ability which requires shared representation and mirror neurons, and will help children make appropriate reactions (Kosonogov et al., 2015; Lamm and Majdandžić, 2015).

No difference in reaction time to and eye movement on sad, angry and happy faces was found. This is noteworthy because previous research has found that angry facial expressions would be detected faster, and elicited fewer fixations and shorter total fixation duration than happy, sad, and neutral ones (LoBue and Larson, 2010; Hunnius et al., 2011). An explanation for the inconsistency is that, as the length of task had to be limited for young children, each emotion was presented only in four trials; therefore the means of behavioral indices were less reliable. Other methodological differences should also be taken into consideration. Like LoBue et al. (2014), we presented an array of four pictures in a trial, but they were positioned in a cross arrangement rather than a matrix and were equally close to the center. Moreover, the participants were 5- to 6- year old children and the facial stimuli were designed in cartoon format. Additionally, there was no time pressure for the visual search task. ANOVAs on standard deviations in both reaction time and the indices of eye tracking found similar patterns of results (with pain showing the smallest *SD*s), which suggested that with just a few observations, how children perceived angry, happy and sad faces might not have been so stabilized as with the painful ones. An explanation tells that pain was perceived in a more stable way because it was evolutionarily crucial, and each individual should have his or her own well-established ways in processing painful stimuli.

In order to investigate whether children would perceive painful faces as indeed showing a higher intensity of pain than other expressions, what facial clues children would use and what role empathy played in the process, Experiment 2 was done, whereby we narrowed our focus on the negative facial expressions, namely sad, angry and painful.

## Experiment 2

### Participants

Forty-six 5- and 6-year-old children who did not take part in Experiment 1 were recruited from a local kindergarten. According to the kindergarten’s official records, these children were normally developing and showed no signs of mental disease. This experiment was approved by the Ethics Committee of the School of Psychological and Cognitive Sciences at Peking University. In accordance with the Declaration of Helsinki, we provided parents of each participant with a written description of the experiment before it began. All parents stated in written informed consent that they allowed their child to participate. Four were excluded because they failed to meet the same data quality criteria in Experiment 1, resulting in a sample of 42 (*M*_age_ = 69.20 months, *SD*_age_ = 5.49; 22 males) for analysis. There was no difference in gender composition between the two groups [χ^2^(1, *N* = 42) = 0.10, *p* > 0.05].

### Design and Material

This experiment employed a 2 (Empathy: high, low) × 3 (Facial expression type: painful, sad, angry) two-factor mixed experimental design.

Stimuli for Experiment 2 were eight sets of faces chosen from the collection prepared for Experiment 1, less happy ones.

### Apparatus and Measures

Equipment used was the same as in Experiment 1.

**FIGURE 6:**
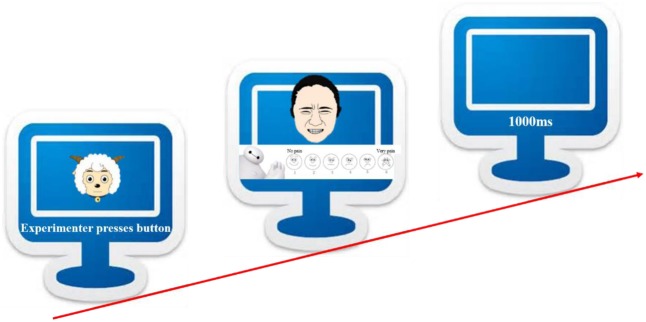
Pain evaluation task diagram.

The experimental task was a pain evaluation task (see **Figure 6**), adapted from Deyo et al. (2004) paradigm. In order to make the evaluation more manageable for children, we used the FACES scale, ranging from 0 to 10 (Wong and Baker, 1988), which had been validated by many studies (Yu et al., 2009; Garra et al., 2013). Participants were told to evaluate the intensity of pain seen in a facial expression of one of the three types plus neutrality, from 1 (not at all) to 6 (very much), and their ratings would be transformed to 0–2–4–6–8–10 for analysis. Eight neutral faces and their 24 emotional morphs would be presented one at a time, yielding a session of 32 trials in a preset random order. The pain intensity of an emotional facial expression was obtained by subtracting the score of its neutral prototype from its raw score. For example, a score of 8 on a painful face would be adjusted to 7 if the neutral face from which the painful one was morphed was rated 1.

### Procedure

Participants were invited to complete the empathy questionnaire as in Experiment 1, and then the pain intensity rating task. Participants were instructed that the screen would first present a fixation picture, then simultaneously present a facial expression (painful, sad, angry, or neutral) and a pain evaluation scale in the form of a row of face icons. The aim was to ensure that the children would stay attentive to pain (Saarela et al., 2007). There was no time limit and the next trial would begin 1s after the participant made a response. Eye movement was recorded the same way as in Experiment 1, and we divided the facial expression into four parts (sub-AOIs) for further analysis: forehead, eyes (include eye brows), nose, and mouth.

### Results

**FIGURE 7:**
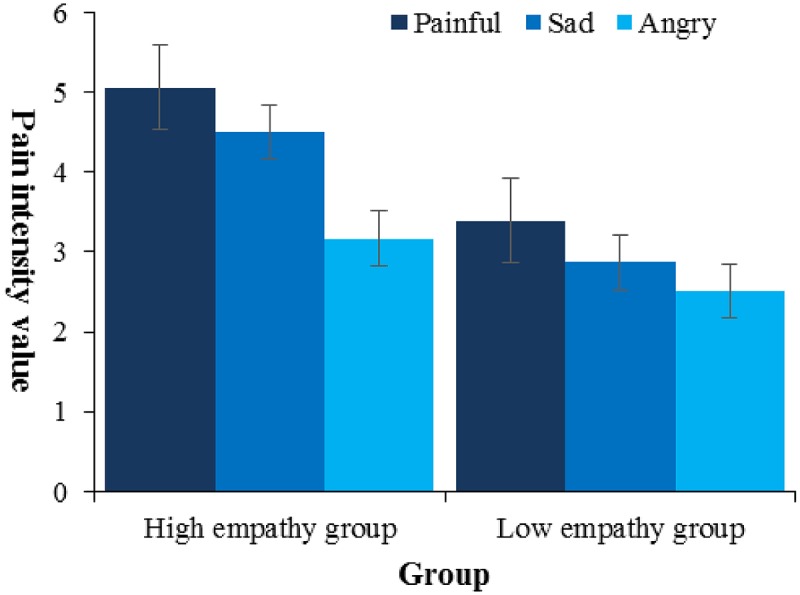
Pain intensity rating scores by type of emotion and empathy.

**FIGURE 8:**
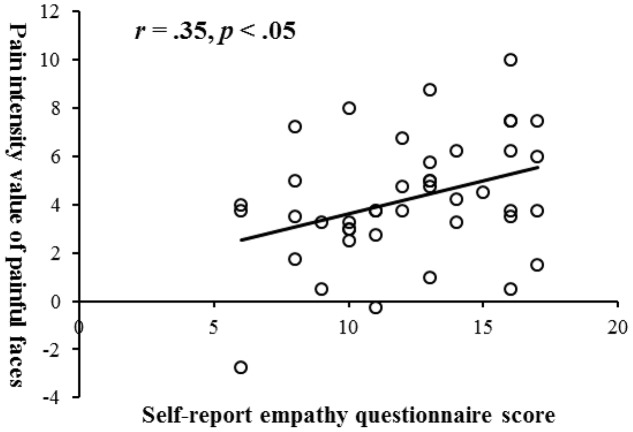
Scatterplot for the correlation between empathy and pain intensity value of painful facial expression with linear regression line.

The questionnaire yielded a Cronbach’s α of 0.78. Subsequently, a 2 × 3 ANOVA evaluated the variation in pain intensity rating associated with level of participant’s empathy and type of facial expression (**Figure 7**). There were two main effects of facial expression type [*F*(2,80) = 9.13, *p* < 0.001, $\eta_{p}^{2}$ = 0.19] and empathy [*F*(1,40) = 6.02, *p* < 0.05, $\eta_{p}^{2}$ = 0.13]. Follow-up pairwise comparisons showed that, first, painful faces (*M* = 4.23, *SE* = 0.38) were evaluated as showing an equal intensity of pain to sad faces (*M* = 4.19, *SE* = 0.24), both of them higher than angry (*M* = 2.84, *SE* = 0.24, *p*s < 0.01); second, high empathy group (*M* = 4.24, *SE* = 0.28) gave higher evaluation than low empathy group (*M* = 3.26, *SE* = 0.28, *p* < 0.05). By-participant correlation analysis (**Figure 8**) showed that only the rating scores of pain intensity seen in the painful faces, not in the sad (*r* = 0.23, *p* > 0.05) or angry faces (*r* = 0.24, *p* > 0.05), were positively related to the questionnaire score of empathy (*r* = 0.35, *p* < 0.05).

**FIGURE 9:**
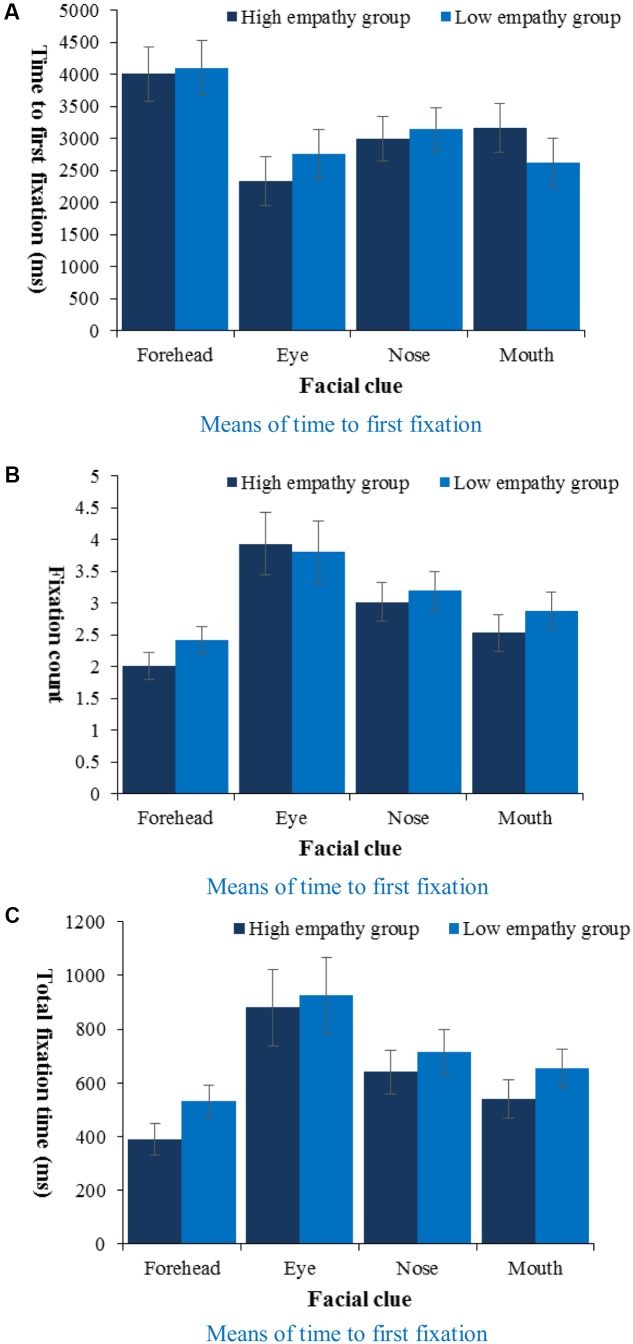
Means of time to first fixation **(A)**, fixation count **(B)**, and total fixation duration **(C)** on painful faces during pain rating task by facial clue and empathy.

To analyze the eye-tracking patterns reflecting how children took advantage of facial clues to evaluate pain intensity, we separately analyzed data in the 8 trials with a painful face. In order to investigate the effects of facial clue, a series of 2 (Empathy: high, low) × 4 (Facial clue: forehead, eyes, nose, mouth) mixed-design ANOVAs on three indices was employed (**Figure 9**). On all indices, only facial clue had a main effect. On time to first fixation [*F*(3,120) = 7.46, *p* < 0.001, $\eta_{p}^{2}$ = 0.16], follow-up pairwise comparison showed that children would fixate onto eyes (*M* = 2547.81 ms, *SE* = 270.33), nose (*M* = 3064.76 ms, *SE* = 243.35) and mouth (*M* = 2892.79 ms, *SE* = 268.41) each more quickly than forehead (*M* = 4053.12 ms, *SE* = 299.69, *p*s < 0.05). On fixation count [*F*(3,120) = 9.47, *p* < 0.001, $\eta_{p}^{2}$ = 0.19], follow-up pairwise comparison showed that children would fixate on eyes (*M* = 3.86, *SE* = 0.35, *p*s < 0.05) more times than forehead (*M* = 2.22, *SE* = 0.15) and mouth (*M* = 2.70, *SE* = 0.21), and fixate on nose (*M* = 3.11, *SE* = 0.21) and mouth more times than forehead (*p*s < 0.05). On total fixation duration [*F*(3,120) = 8.85, *p* < 0.001, $\eta_{p}^{2}$ = 0.18], follow-up pairwise comparisons showed that children would fixate for a longer time on eyes (*M* = 903.50 ms, *SE* = 100.60, *p*s < 0.05) than forehead (*M* = 459.90 ms, *SE* = 42.02) and mouth (*M* = 596.84 ms, *SE* = 50.45), and fixate for a longer time on nose (*M* = 677.56 ms, *SE* = 58.01) and mouth than forehead (*p*s < 0.05). Then, we did twelve by-subject correlation analyses between pain rating and the three eye tracking indices on the face’s four clues, and found that time to first fixation on eyes (*r* = -0.33, *p* < 0.05), fixation count on mouth (*r* = -0.47, *p* < 0.01), total fixation duration on mouth (*r* = -0.46, *p* < 0.01) were negatively related to pain rating.

In order to find the most predictive index, a hierarchical regression was done (see **Table 2**). The regression results showed that the best predictor was self-reported empathy score. In other words, the higher an individual was in empathy, the higher she/he would rate the pain observed.

**Table 2:** Hierarchical regression on pain rating (with Enter method, *N* = 42).

Variable	*B*	β	*t*	*p*
Step 1				
Self-reported empathy score	0.27	0.35	2.39	<0.05
Step 2				
Self-reported empathy score	0.23	0.29	2.21	<0.05
Time to first fixation on eyes	0.00	-0.20	-1.50	>0.05
Fixation count on mouth	-0.64	-0.34	-1.10	>0.05
Total fixation duration on mouth	-0.00	-0.08	-0.27	>0.05

*Step 1: *R*_*change*_^*2*^ = 0.13; *R*_*adjust*_^*2*^ = 0.10; Step 2: *R*_*change*_^*2*^ = 0.24; *R*_*adjust*_^*2*^ = 0.30 (*p*s < 0.05).*

### Discussion

This study found that children fixated on eyes and mouth more quickly, more frequently and for longer times. The findings implied that children were most sensitive to these parts, and they might be the most important clues for judging the pain intensity in facial expressions. Previous studies have suggested they are also the most important facial clues for the decoding of other emotional expressions in adults and children (Eisenbarth and Alpers, 2011; Guarnera et al., 2015). These results agreed with previous studies to a certain extent as they suggested that visual perception of brows, orbits, nose, and eyes were the best predictors for the judgment of pain in facial expression (Williams, 2002; Prkachin, 2011).

A potential explanation why children relied primarily on mouth and eyes is offered. Facial expressions have many facial clues, but children’s limitations in their cognitive capacities mean that they can only use one or two major ones (Eisenbarth and Alpers, 2011; Cowan et al., 2014) to evaluate the pain intensity, which is an economic way for them. The study by LoBue and Larson (2010) showed that infants may always identify the angry facial expression with the same clue, V-shaped brows, which carry the most useful information. Deyo et al. (2004) found that the ability to take advantage of facial clues was related to age, and children aged 5 and 6 years performed poorly, with brows and mouth being the most useful facial clue for them to rate pain.

Importantly, Experiment 2 agrees with our results from Experiment 1 because both suggested that children high in empathy had a lower threshold in the perception of pain. Children’s rating of pain intensity in observed faces was related to their empathy measured by a self-reported questionnaire, which extended the finding of Allen-Walker and Beaton (2015) that averaged ratings of intensity across six emotions in Facial Expressions of Emotion: Stimuli and Tests was significantly correlated with participants’ Empathy Quotient. Previous research in adults showed that people scoring higher in empathy wound exhibit higher pain-related brain activity (Singer et al., 2004; Saarela et al., 2007), and observers’ brain activity was in turn related to the rated intensity of pain shown (Saarela et al., 2007). Hence, empathy and evaluation of pain might share common neural basis, which could have developed considerably by preschool years.

It was unexpected, however, that children’s evaluations of pain intensity for sad and painful facial expressions did not differ. Given the accuracy of children’s self-report about pain (Spagrud et al., 2003), one possible explanation is, as Kappesser and Williams (2002) proposed, that patterns of FAUs and patterns in the messages conveyed by the two expressions were the same. Additionally, the face icons in the FACES scale in this study could be more easily perceived as sad than as painful facial expressions. As a result, they could have distracted children and interfered with their judgments.

## General Discussion

Taken together, these findings showed us a picture that children’s empathy had a positive influence on their perception and an “amplifying” effect on the evaluation of pain in facial expression, and pain may be distinct from the other facial expressions in terms of eye-tracking indices. The present study provided evidence for a visual profile for facial expression of pain – faster orientation, shorter fixation duration and less fixation count. It may reflect the natural implication of pain – threat (Yamada and Decety, 2009; Todd et al., 2016), which may be imprinted through evolution and anchored in facial clues. Other similar facial expressions, e.g., threat-related expressions (such as angry and fearful ones), also elicits the same visual profile (Hunnius et al., 2011), but they are more closely associated with social threats (Fox et al., 2000; Lobue et al., 2016), and not a major sign for a life-threatening situation, possibly because these expressions differ in the later stages of visual processing. Some researchers suggested that attention might be more biased toward painful facial expressions than angry ones at the beginning (up to 1000 ms), and became increasingly less so thereafter (Priebe et al., 2015). Also notably, pain, like sadness, will induce personal distress in the observer (Luo et al., 2015). But facial expression of pain conveys more information regarding the physical environment.

Moreover, most researchers divided empathy into two facets, cognitive empathy and affective empathy (Shamay-Tsoory et al., 2009; Wu et al., 2012). From the developmental perspective, humans are born with the primary component of empathy, emotional contagion (Preston and de Waal, 2002), which mainly reflects the affective facet; and by 36 months will the ability to understand emotions develop (Decety, 2010), which mainly reflects the cognitive facet. In view of the aforementioned facts, we propose that cognitive empathy and affective empathy may play different roles in the feeling and perception of pain. A previous study by us in adults (Yan et al., 2016) has found that from processing of attention to making a painful facial expression, empathy would only influence the attentional maintenance stage, but here no influence of empathy was found on this stage in children. This is explainable as in children, the affective component of empathy develops earlier than the cognitive component and functions as the dominant one, and their immature cognitive abilities makes it hard for them to regulate their emotional reaction. It is also noticeable that although Experiment 1 showed that painful faces attracted children’s attention most quickly, and Experiment 2 showed that only the rating of pain intensity seen in the painful faces were positively related to empathy, the superiority of high empathy in child on their behavioral and perceptual response was manifested across emotions. First, this is consistent with previous findings that empathy facilitated the processing of more than one type of faces (Dimberg et al., 2011; Choi and Watanuki, 2014). Second, the affective component of empathy was affective arousal or emotional contagion (Preston and de Waal, 2002; Decety, 2010). This component would help decrease our threshold for emotional stimuli, and it would affect the processing of all types of facial expression rather than a specific one.

Some researchers theorized that, because painful stimuli are associated with a potential threat, and perception of others’ pain alone does not automatically activate an empathic process, a threat-detection system appears to be activated first, with a possibly general aversive response in the observer, instead of an empathic response (Ibáñez et al., 2011). Since threats might mean more serious harm to children than to adults, as a result of evolution, to avoid perceived threat might be given a higher priority in children, and that might explain why we failed to found any relationship between empathy and later attentional processing in Experiment 2. Yet other researchers suggested that observing others’ pain would trigger empathy for their pain (Decety and Lamm, 2006).

## Strengths, Limitations, and Conclusion

Future studies can improve on the several limitations presented in our study. First, it has yet to be answered whether the cognitive or the affective facet of empathy influenced children’s performance. Larger sample sizes and more refined measurements of empathy would allow for analysis of empathy by facet. Second, in order to get the full picture of development, future research could cover people of all ages (Deyo et al., 2004), which is the best way to know how empathy and pain work together. Third, we suggest researchers solve the potential measurement issue in pain rating task by precluding potential confusion caused by sad faces used in the instrument and using a wider range of methodologies, such as the FACES pain scale (Hicks et al., 2001). Fourth, further research should probably consider the influence of IQ and language level to make the findings more reliable. Finally, the format of material should be taken into consideration. In cartoonized facial expressions used in the study, many details essential to real faces like texture were absent. Although we had removed or neutralized common gender clues, there might still be individual differences in how people perceived the gender of the faces. While in this way cartoons made it easier for young children to access the emotional information (Kendall et al., 2015), the ecological validity of the findings could be harmed because the cartoons differed substantially from real faces seen daily.

To conclude, the current study suggested that 5- to 6-year-old children could detect painful facial expression in the shortest time and with the lowest visual effort, and individuals high in empathy performed better in the search than those low in empathy. In addition, 5- to 6-year-old children primarily relied on eyes and mouth as clues to evaluate pain intensity of a painful facial expression. The observed pain was perceived to be stronger by raters higher in empathy.

## Ethics Statement

All procedures performed in the study involving human participants were conducted in accordance with the ethical standards of the institutional and national research committee and with the 1964 Helsinki declaration and its later amendments or comparable ethical standards. Informed consent was obtained from all participants included in the study.

## Author Contributions

ZY and YS contributed to the conception and design of the work. ZY collected and analyzed the data. ZY, MP, and YS contributed to the writing of the manuscript.

## Conflict of Interest Statement

The authors declare that the research was conducted in the absence of any commercial or financial relationships that could be construed as a potential conflict of interest.
